# Recombinant Porcine 12-*Lipoxygenase Catalytic Domain*: Effect of Inhibitors, Selectivity of Substrates and Specificity of Oxidation Products of Linoleic Acid

**DOI:** 10.3390/foods11070980

**Published:** 2022-03-28

**Authors:** Jiamei Xu, Yu Liu, Jingjing Ma, Pengpeng Li, Zhiming Geng, Daoying Wang, Muhan Zhang, Weimin Xu

**Affiliations:** 1 School of Food and Biological Engineering, Jiangsu University, Zhenjiang 212013, China; j15751018163@163.com (J.X.); ly786180331@163.com (Y.L.); 2 Institute of Agro-Products Processing, Jiangsu Academy of Agricultural Sciences, Nanjing 210014, China; jingjingma2017@163.com (J.M.); daoyingwang@yahoo.com (D.W.); zhangmh35@gmail.com (M.Z.); weiminxu2002@aliyun.com (W.X.); 3 Jiangsu Collaborative Innovation Center of Meat Production and Processing, Quality and Safety Control, Nanjing 210095, China

**Keywords:** *Lipoxygenase*, inhibitors, substrates, oxidation products, linoleic acid, porcine

## Abstract

*Lipoxygenase (LOX)* is a major endogenous enzyme for the enzymatic oxidation of lipids during meat storage and meat product manufacturing. In the present work, some characteristics, i.e., effects of inhibitors, selectivity of substrates and specificity of oxidation products, were studied using recombinant porcine 12-*lipoxygenase catalytic domain* (12-LOXcd). Several familiar inhibitors were found inhibit the activity of recombinant porcine 12-LOXcd;nordihydroguaiaretic acid demonstrated the strongest inhibitory effect. The enzyme could oxygenate common polyunsaturated fatty acids, and showed the highest affinity to linoleic acid (LA), followed by arachidonic acid (AA), linolenic acid (LN) and docosahexaenoic acid (DHA). Under the action of porcine 12-LOXcd, LA was oxidized into four hydroxyoctadecadienoic acid (HODE) isomers, i.e., 13-*Z,E*-HODE, 13-*E,E*-HODE, 9-*Z,E*-HODE and 9-*E,E*-HODE. Variation of pH not only affected the yield of LA oxidation products, but also the distribution of HODE isomers. These results indicated that endogenous LOX activity and LOX-catalyzed lipid oxidation can be regulated during meat storage and meat product manufacturing.

## 1. Introduction

Lipids oxidation is one of the most important biochemical reactions in the processing of meat and meat products; it occurs in the whole process from the raw meat to final products [1]. The oxidation of lipids manifests in color changes in raw meat and the formation of spoiled flavors and/or odors in meat products. It is considered as a major determinant of meat quality [2,3]. This process can be induced by free radicals or nonradical reactive oxygen species, and can be catalyzed by some endogenous enzymes (e.g., *lipoxygenase*) as well. Nonenzymatic and enzymatic lipid oxidations usually take place simultaneously in meat processing or meat products. For cured meat products, lipid oxidation by *lipoxygenase* plays a significant role in the development of characteristic flavor; it is dependent on adequate physical–chemical conditions (e.g., temperature and water activity) and the long period of time required for cured meats manufacturing [4].

Lipoxygenase (linoleate: oxidoreductase, EC 1.13.11.12) is a group of dioxygenases containing nonheme ion which catalyzes the regio- and stereo-specific dioxygenation of polyunsaturated fatty acids (PUFAs) to conjugated unsaturated fatty acid hydroperoxides [5]. Lipoxygenase (LOX) is widely distributed in plants, animals and microorganisms. The oxidation of lipids has a direct impact on the quality, especially on the flavor, of meat products [6]. Quite a lot of studies have been carried out on endogenous LOX in the manufacture of meat products, including extraction of the enzyme, the effects of physical–chemical factors on activity, oxidation products of PUFAs and contribution to flavor [5,6,7]. Compared with LOX in plants which have been studied in depth, only a couple of LOX in animals, i.e., isolated from pig, chicken and fish, have been partially characterized [7,8,9]. Due to the huge difficulty in the purification of LOX, there is still a lack of deeper study on LOX in animal tissue, e.g., selectivity of substrates, distribution of oxidation products of PUFAs etc.

In addition to being involved in the formation of flavor, the oxidation of lipids may give rise to the off-odors [10], which impair the sensory quality of the meat. Some secondary products of oxidation, e.g., hydroxynonenal and hydroxyoctadecadienoic acid, have been connected with a series of human chronic diseases [11,12]. Therefore, deeper study of the enzymatic properties of LOX in animal tissue would help us to better understand the role played by endogenous LOX during the manufacture of meat products. Such study would pave the way for further research into the mechanisms behind LOX-catalyzed oxidation of lipids in the manufacture of meat products, as well as the implications to human health.

In a previous work, recombinant porcine 12-*lipoxygenase catalytic domain* (12-LOXcd) was expressed in *Escherichia coli* (*E. coli*) and purified, and its fundamental enzymatic properties were studied [13]. The objectives of this paper were: (1) to investigate the effect of inhibitors on recombinant porcine 12-LOXcd, (2) to observe the substrate selectivity of 12-LOXcd, and (3) to study the distribution of the oxidation products of LA by 12-LOXcd and evaluate the effects of pH on that distribution.

## 2. Materials and Methods

### 2.1. Materials

Linoleic acid (LA), linolenic acid (LN), arachidonic acid (AA), docosahexaenoic acid (DHA) and nordihydroguaiaretic acid (NDGA) were obtained from Sigma Chemical Company (Shanghai, China). Phenidone was purchased from Shanghai Yuanye Biotechnology Company Ltd. (Shanghai, China). Caffeic acid, iodoacetamide, and β-mercapto-ethanol were purchased from Shanghai Aladdin Biochemical Technology Company Ltd. (Shanghai, China). Finally, 13-*Z,E*-hydroxy-9,11-octadecadienoic acid (13-*Z,E*-HODE), 13-*E,E*-hydroxy-9,11-octadecadienoic acid (13-*E,E*-HODE), 9-*E,Z*-hydroxy-10,12-octadecadienoic acid (9-*Z,E*-HODE), and 9-*E,E*-hydroxy-10,12-octadecadienoic acid (9-*E,E*-HODE) were provided by Cayman Chemicals (Ann Arbor, MI, USA).

The organic solvents used for chromatographic separation, including *n*-hexane, 2-propanol and acetic acid, were obtained from Merck (Darmstadt, Germany). All other reagents were of analytical grade.

### 2.2. Preparation of Recombinant Porcine 12-LOXcd

Recombinant porcine 12-LOXcd was expressed in *E. coli* and purified as described previously [13]. In brief, the catalytic domain of 12-LOXcd was obtained by sequence analysis and polymerase chain reaction (PCR) amplification. The coding sequence of 12-LOXcd was constructed into an inducible expression vector and expressed in *E. coli* induced by isopropyl β-*D*-1-thiogalactopyranoside (IPTG). The recombinant protein was purified by Ni-NTA affinity chromatography and Superdex 200 gel filtration chromatography.

Protein was determined by the Bradford method using bovine serum albumin as standard [14].

### 2.3. Determination of Activity of Recombinant Porcine 12-LOXcd

The substrate solution preparation: First, 280 mg of LA and 360 µL of Tween 20 were added to 10 mL of deoxygenated water, followed by the addition of 1 M NaOH until the LA was totally dissolved. The solution was adjusted to pH 9.0 with 1 M HCl and deoxygenated water was added to obtain a total volume of 100 mL. Finally, the solution was flushed with nitrogen and kept under an oxygen-free atmosphere at −20 °C.

The determination of porcine 12-LOXcd activity was carried out on a UV6000 UV-Vis spectrophotometer (Mapada, Shanghai, China) at 20 °C. Porcine 12-LOXcd activity was reflected by an increment in absorbance at 234 nm resulting from the formation of conjugated double bonds in the primary oxidation products of LA over a certain period of reaction time. The reaction system was composed of 200 µL of the substrate solution, 100 µL of enzymatic solution, and 1.7 mL of 50 mM citrate buffer (pH 5.5). In the control reaction system, the same volume of citric acid buffer was used to replace the enzyme solution. The reaction lasted for 1 min. One unit (U) of porcine 12-LOXcd activity was defined as an increment of 0.001 in absorbance at 234 nm per minute.

### 2.4. Kinetics of Recombinant Porcine 12-LOXcd Oxidation

The kinetics properties were investigated under the same conditions as those for determination of porcine 12-LOXcd activity, as described in Section 2.3, with substrate concentrations ranging from 0.2 mM to10 mM. The Michaelis constants (*K*m) and the maximum velocities (*V*max) of the substrates (i.e., LA, LN, DHA and AA) were calculated by using GraphPad Prims 5.01 (GraphPad Software, Inc., San Diego, CA, USA).

### 2.5. Oxidation of LA by Porcine 12-LOXcd and Extraction of Oxidation Products

The reaction system for the oxidation of LA by porcine 12-LOXcd was composed of 100 µL of the enzyme solution (containing 0.05 mg protein) and 2 mL of acetate buffer (25 mM, pH 5.5–7.5) containing 10 mM LA and 1.8 µL of Tween 20. The reaction was carried out at 30 °C for 60 min. The reaction mixture was adjusted to pH 3 with 1 M HCl, followed by reduction via the addition of 100 mg of potassium borohydride. After 0.5 h, the reaction mixture was extracted with chloroform/methanol (3:1, *v*/*v*) and evaporated to dryness under a stream of nitrogen. The dried extract was dissolved in 400 µL of n-hexane.

### 2.6. Normal Phase High Performance Liquid Chromatography (NP-HPLC) Analysis of Oxidation Products of LA by Porcine 12-LOXcd

The NP-HPLC analysis of oxidation products of LA by porcine 12-LOXcd was carried out on an e2695 HPLC system (Waters, Milford, MA, USA) coupled with a 2998 photodiode array detector (Waters, Milford, MA, USA) and an Absolute SiO_2_ column (250 × 4.6 mm, 5 µm). The samples were eluted at 1.0 mL/min at 30 °C with n-hexane/2-propanol/acetic acid (983:16:1) as the mobile phase. The analytes were monitored at 234 nm, and detected qualitatively by comparing retention times with corresponding standards and quantitatively by an external standard method.

### 2.7. Statistic Analysis

All experiments and analytical tests were performed in triplicate and data are expressed as mean ± SD. One-way analysis of variance (ANOVA) was carried out with SPSS 17.0 for Windows (Chicago, MI, USA) with a significance level of *p* < 0.05. All graphs and calculations were created using the Origin Pro 8.1 SR3 software package (MA, USA).

## 3. Results and Discussion

### 3.1. Effects of Inhibitors on the Activity of Recombinant Porcine 12-LOXcd

A wide range of compounds from different sources have been reported to inhibit lipoxygenases. In the present paper, the inhibiting activities of several compounds on porcine 12-LOXcd were investigated. The results are listed in Table 1.

Inhibition was evaluated by the residual activity of porcine 12-LOXcd, which was obtained by comparison between activities with and without the inhibitor. NDGA (nordihydroguaiaretic acid) and caffeic acid (3,4-dihydroxycinnamic acid) are natural phenolic compounds with the ability to reduce ferric to catalytically inactive ferrous [15,16]. These compounds were also found to be inhibitors of a wide range of lipoxygenases [17,18]. In the present study, both compounds exhibited strong inhibitions of porcine 12-LOXcd. NDGA, which has been accepted as a specific inhibitor of LOX [19], was the strongest among the tested compounds. Phenidone is a pyrazoline derivative which has been regarded as a dual inhibitor of cyclooxygenase and lipoxygenase [20,21]. The inhibition mechanism involves the irreversible binding of a phenidone metabolite to a concomitant oxidized amino acid residue in the lipoxygenase [21]. Phenidone was found to be a potent inhibitor of porcine 12-LOXcd with the second strongest observed inhibition in the present paper. Sulfhydryl groups and cysteine bridges play important roles in preserving lipoxygenase activity [22,23]. Iodoacetamide and β-mercaptoethanol inhibit lipoxygenases by reacting with sulfhydryl groups and breaking up cysteine bridges in the enzymes, respectively. In the present work, these compounds exerted relatively weaker inhibitions of porcine 12-LOXcd. In general, all the tested compounds demonstrated strong inhibitions of porcine 12-LOXcd at concentrations of µM or mM, which is in agreement with results of a study on lipoxygenase extracted from pig muscle [23].

### 3.2. Substrate Selectivity of Recombinant Porcine 12-LOXcd

For the substrate selectivity of porcine 12-LOXcd, four free PUFAs (i.e., LA, LN, AA and DHA) were employed to examine the effect of substrate concentration on enzyme activity. The enzyme activity followed classical Michaelis-Menten kinetics when recombinant porcine 12-LOXcd was incubated with the substrates at various concentrations. The *K*m and *V*max values were estimated based on Lineweaver-Burk plot analysis, and are summarized in Table 2.

The obtained *K*m value for LA was 149 µM, which was higher than that of LOX (68 µM) from Chinese crossbreed pig muscle [7], and lower than that of LOX (280 µM) from Iberian pig muscle [23]. Compared with *K*m values for LA of other mammalian LOX, e.g., 12.1µM of rabbit reticulocyte LOX and 31.2µM of porcine leukocyte LOX [24], the obtained *K*m value was significantly higher. Such differences might be ascribed to different sources of the enzyme, as well asvariations in the procedures used to assay the enzymatic activity [7,23]. Based on *V*max/*K*m, efficacy as a substrate of recombinant porcine 12-LOXcd was the greatest for LA, followed by AA, LN, and DHA. Therefore, LA was the optimum substrate for recombinant porcine 12-LOXcd, which was also in agreement with our understanding that LA is the most abundant PUFA in mammalian tissues [25].

### 3.3. Specificity of Oxidation Products of LA by Recombinant Porcine 12-LOXcd

There are multiple carbon–carbon double bonds in PUFAs, which yield oxidation products composed of series of isomers when PUFAs are oxidized. In nonenzymatic oxidation, PUFAs are usually oxidized into isomers with the same quantity [26], while the specificity of isomers depends on the origin of LOX in LOX-catalyzed oxidation [27,28]. Therefore, the specificity of products from LOX-catalyzed lipid oxidation should be further studied to understand the evolution of lipid oxidation patterns in this complicated system [26]. In the present paper, the product specificity of oxidation by porcine 12-LOXcd with LA as the substrate was investigated. Hydroperoxyoctadecadienoic acids (HPODEs), i.e., the primary products of LA oxidation by porcine 12-LOXcd, were reduced to hydroxyoctadecadienoic acids (HODEs) by potassium borohydride. The reduction transforms chemically-active hydroperoxides into relatively stable hydroxides without changing their isomerization characteristics; the latter can then be conveniently and precisely detected by HPLC-based methods [23,29].

**Figure 1:**
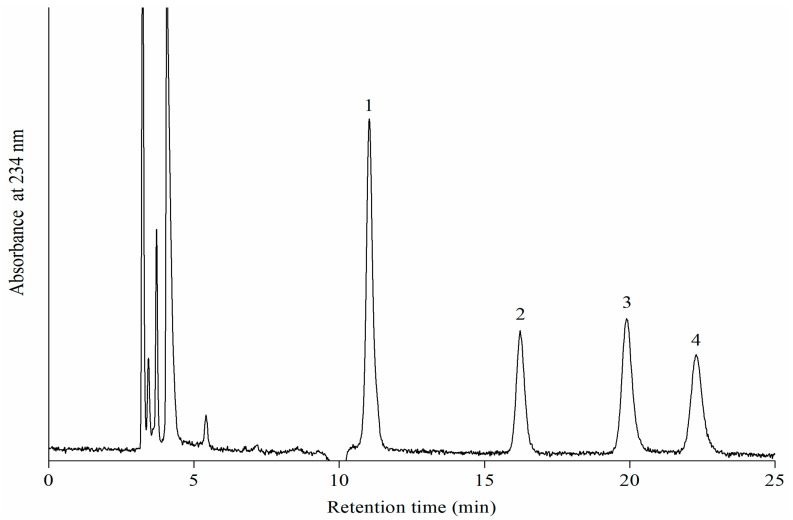
NP-HPLC chromatogram of reduced hydroperoxides from LA oxidation by recombinant porcine 12-LOXcd, peaks assignments: (1) 13-*Z,E*-HODE; (2) 13-*E,E*-HODE; (3) 9-*Z,E*-HODE; (4) 9-*E,E*-HODE.

Under the action of porcine 12-LOXcd, LA was oxidized into four HODE isomers, i.e., 13-*Z,E*-HODE, 13-*E,E*-HODE, 9-*Z,E*-HODE, and 9-*E,E*-HODE. All HODEs were identified from the coelution of the corresponding standards, and the profiles of the oxidation product isomers were in good agreement with those reported for lipoxygenases from animals [23,30], microorganisms [29], and plants [31]. Figure 1 displays a NP-HPLC chromatogram of HODEs from LA oxidation by recombinant porcine 12-LOXcd. The ratios of 13-*Z,E*-HODE, 13-*E,E*-HODE, 9-*Z,E*-HODE and 9-*E,E*-HODE in an incubation system at pH 5.5 were found to be 0.35, 0.23, 0.20 and 0.22 respectively, which was similar to the results from a study by Gata et al. [23], who purified LOX in Iberian pig muscle and investigated the distribution of oxidation product isomers of LA.

### 3.4. The Impacts of pH on Yield and Specificity of Oxidation Products of LA by Recombinant Porcine 12-LOXcd

For some meat products, e.g., cured meats, manufacturing usually requires a long period of time, and endogenous enzymes including LOX may remain active throughout the process due to the mild physical–chemical conditions involved. In general, pH may vary within a certain range during the manufacture of cured meat products [7]. In this paper, the impact of variations in pH on the yield and distribution of LA oxidation products (HODEs) by porcine 12-LOXcd was investigated. To negate the contribution of nonenzymic oxidation, parallel analyses were conducted on reaction mixtures incubated with heat-denatured enzymes and LA.

Table 3 lists the results of total HODEs and shares of HODE isomers in incubation systems with pH ranging from 5.5 to 7.5. The total HODEs, i.e., the sum of four HODE isomers in the incubation system, was found to reach the top value at pH 6.0; it then declined with increasing of pH value. Therefore, the optimum pH for the production of HODEs by porcine 12-LOXcd was 6.0, which was in agreement with the enzymatic characteristics reported in a previous work [13]. This result was similar to the optimum pH for LOX from other sources [32].

**Figure 2:**
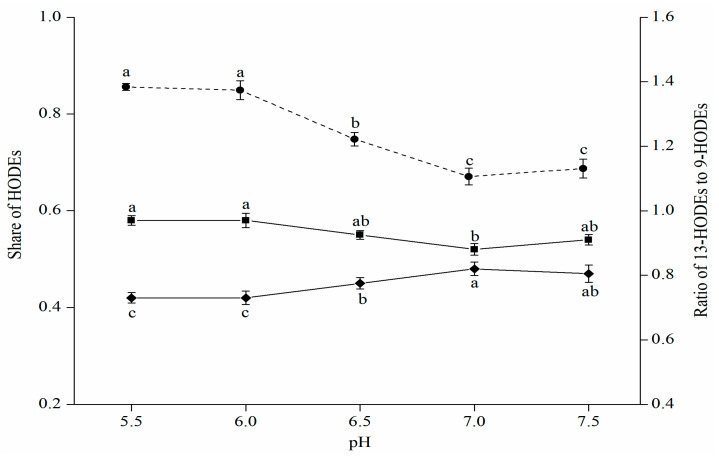
Variations of shares of 13-HODEs (—■—), 9-HODEs (—◆—) and ratio of 13-HODEs to 9-HODEs (--●--) with pH increasing from 5.5 to 7.5. Values with different letters in the same line are significantly different (*p* < 0.05).

The impact of pH variation on the distributions of HODE isomers in LA oxidation by porcine 12-LOXcd was also investigated. Figure 2 displays variations in the shares of 13-HODEs and 9-HODEs, and the ratio of 13-HODEs to 9-HODEs with pH increasing from 5.5 to 7.5. In the first and the last pH variation ranges, i.e., 5.5 to 6.0 and 7.0 to 7.5, the shares of 13-HODEs and 9-HODEs remained almost unchanged. However, with increasing the pH from 5.5 to 7.0, the share of 13-HODEs (13-*Z,E*-HODE + 13-*E,E*-HODE) declined significantly (*p* < 0.05), while that of 9-HODEs (9-*Z,E*-HODE + 9-*E,E*-HODE) increased significantly (*p* < 0.05), which resulting in the ratio of 13-HODEs to 9-HODEs also decreasing significantly (*p* < 0.05), i.e., from 1.38 to 1.10.

Studies on plant LOX have indicated that pH alterations have a significant impact on the distribution of oxidation product isomers [30]. With this in mind, soybean LOX and potato LOX were employed to prepare standard 13-HODE at pH 9.0 and 9-HODE at pH 6.0, respectively, in early studies on the characterization of LA oxidation products by LOX [23]. There is still scarce information in the literature on the impact of pH on the distribution of oxidation products by animal LOX.

The impact of pH alteration on the distribution of oxidation products is generally ascribed to its dual consequences for enzymatic oxidation by LOX [30]: (1) pH alteration changes the degree of dissociation of the fatty acid substrate, which determines whether the substrate is in a carboxylic group or in an uncharged molecule; (2) pH alteration modifies the degree of dissociation of amino acid side chains, which may result in changes to the substrate orientation. In the present study, pH varied in the range from 5.5 to 7.5. Under these conditions, LA would have always been in an uncharged form due to its p*K*a of 9.24 [33]. Therefore, pH alteration might have changed the substrate orientation by modifying the degree of dissociation of specific amino acid(s) in porcine 12-LOXcd, which resulted in the variation of the distribution of oxidation products.

## 4. Conclusions

Recombinant porcine 12-LOXcd could be inhibited by common LOX inhibitors; it was observed that NDGA was the strongest inhibitor. Recombinant porcine 12-LOXcd could oxygenate PUFAs including LA, LN, AA and DHA, with LA being the optimum substrate. LA was oxidized to four HODE isomers by recombinant porcine 12-LOXcd. Variation of pH not only affected the yield but also the distribution of LA oxidation products by recombinant porcine 12-LOXcd. This result indicated that variation of pH might exert an important effect on endogenous LOX catalyzed lipid oxidation during meat storage and the manufacture of meat products.

## Figures and Tables

**Table 1:** Effects of inhibitors on the activity of porcine 12-LOXcd (mean ± SD, *n* = 3) ^1^.

Compound	Concentration	Residual Activity (%)
NDGA	0.03 µM	38.0 ± 1.9 ^c^
NDGA	2.5 µM	3.8 ± 0.4 ^g^
caffeic acid	0.01 mM	57.0 ± 2.3 ^a^
caffeic acid	0.1 mM	4.8 ± 0.1 ^f^
phenidone	0.12 µM	46.0 ± 1.6 ^b^
phenidone	20 µM	6.1 ± 0.5 ^e^
iodoacetamide	0.12 mM	64.0 ± 5.3 ^a^
iodoacetamide	4 mM	6.4 ± 0.5 ^e^
β-mercaptoethanol	0.2 mM	38.2 ± 1.8 ^c^
β-mercaptoethanol	1.4 mM	9.2 ± 0.9 ^d^

^1^ Values with different letters in the same column are significantly different (*p* < 0.05).

**Table 2:** Values of kinetic parameters for recombinant porcine 12-LOXcd (mean ± SD, *n* = 3) ^1^.

Substrate	*K*m (µM)	*V*max (U/mg)	*V*max/*K*m (U/mg/µM)
LA	149 ± 1.2 ^d^	0.019 ± 0.001 ^d^	0.1275 ± 0.0033 ^a^
LN	592 ± 2.7 ^c^	0.031 ± 0.003 ^c^	0.0524 ± 0.0011 ^c^
AA	909 ± 2.0 ^b^	0.076 ± 0.007 ^b^	0.0836 ± 0.0015 ^b^
DHA	8766 ± 5.5 ^a^	0.214 ± 0.011 ^a^	0.0244 ± 0.0012 ^d^

^1^ Values with different letters in the same column are significantly different (*p* < 0.05).

**Table 3:** Variations of ratio of HODE isomers and yield of HODEs with pH ranging from 5.5 to 7.5 (mean ± SD, *n* = 3) ^1^.

pH	13-*Z,E*-HODE	13-*E,E*-HODE	9-*Z,E*-HODE	9-*E,E*-HODE	Total HODEs ^2^
5.5	0.35 ± 0.005 ^b^	0.23 ± 0.005 ^a^	0.20 ± 0.006 ^d^	0.22 ± 0.005 ^a^	4.78 ± 0.14 ^b^
6.0	0.36 ± 0.008 ^b^	0.22 ± 0.007 ^a^	0.22 ± 0.007 ^c^	0.20 ± 0.007 ^b^	5.22 ± 0.09 ^a^
6.5	0.36 ± 0.004 ^b^	0.19 ± 0.005 ^b^	0.24 ± 0.008 ^b^	0.21 ± 0.004 ^ab^	4.62 ± 0.11 ^b^
7.0	0.37 ± 0.006 ^ab^	0.15 ± 0.006 ^c^	0.28 ± 0.006 ^a^	0.20 ± 0.008 ^b^	4.26 ± 0.16 ^c^
7.5	0.40 ± 0.007 ^a^	0.14 ± 0.004 ^c^	0.25 ± 0.009 ^b^	0.22 ± 0.009 ^a^	3.83 ± 0.13 ^d^

^1^ Values with different letters in the same column are significantly different (*p* < 0.05). ^2^ The sum of four HODEs produced in the incubation system (µg).

## Data Availability

Not applicable.

## Abbreviations

AA: arachidonic acid; DHA, docosahexaenoic acid; HODE, hydroxyoctadecadienoic acid; LA, linoleic acid; LN, linolenic acid; *LOX, lipoxygenase;* 12-LOXcd, 12-*lipoxygenase catalytic domain*; NDGA, nordihydroguaiaretic acid; PUFAs, polyunsaturated fatty acids.
